# Susceptibility of Oocytes from Gilts and Sows to Beauvericin and Deoxynivalenol and Its Relationship with Oxidative Stress

**DOI:** 10.3390/toxins13040260

**Published:** 2021-04-06

**Authors:** Eric J. Schoevers, Regiane R. Santos, Bernard A. J. Roelen

**Affiliations:** 1 Farm Animal Health, Department Population Health Sciences, Faculty of Veterinary Medicine, Utrecht University, Yalelaan 104, 3584 CM Utrecht, The Netherlands; ejschoevers@uu.nl; 2 Schothorst Feed Research, P.O. Box 533, 8200 AM Lelystad, The Netherlands; 3 Embryology, Anatomy and Physiology, Department Clinical Sciences, Faculty of Veterinary Medicine, Utrecht University, Yalelaan 1, 3584 CT Utrecht, The Netherlands; b.a.j.roelen@uu.nl

**Keywords:** mycotoxins, oocyte, follicular fluid, cumulus cells, in vitro maturation, swine

## Abstract

Beauvericin (BEA) and deoxynivalenol are toxins produced by *Fusarium* species that can contaminate food and feed. The aim of this study was to assess the effects of these mycotoxins on the maturation of oocytes from gilts and sows. Furthermore, the antioxidant profiles in the oocytes’ environment were assessed. Cumulus-oocyte-complexes (COCs) from gilts and sows were exposed to beauvericin (BEA) or deoxynivalenol (DON) and matured in vitro. As an extra control, these COCs were also exposed to reactive oxygen species (ROS). The maturation was mostly impaired when oocytes from gilts were exposed to 0.02 μmol/L DON. Oocytes from sows were able to mature even in the presence of 5 μmol/L BEA. However, the maturation rate of gilt oocytes was already impaired by 0.5 μmol/L BEA. It was observed that superoxide dismutase (SOD) and glutathione (GSH) levels in the follicular fluid (FF) of gilt oocytes was higher than that from sows. However, the expression of SOD1 and glutathione synthetase (GSS) was higher in the oocytes from sows than in those from gilts. Although DON and BEA impair cell development by diverse mechanisms, this redox imbalance may partially explain the vulnerability of gilt oocytes to these mycotoxins.

## 1. Introduction

Mycotoxins are food and feed contaminants produced by several fungal species. Depending on the dosage and toxin type, feed contamination may impair animal health, production performance, or reproductive function. The negative effects of several mycotoxins on female porcine reproduction has previously been demonstrated in in vitro [1,2,3,4,5,6] and in vivo studies [7,8]. From the studied mycotoxins, zearalenone (ZEN) and alternariol (AOH) simulate estrogenic activity, competing with estrogen receptors and, hence, interfering with oocyte growth [7], maturation [6], embryo development [1,6], and hormonal function of the progeny [8]. Other mycotoxins like deoxynivalenol (DON) and beauvericin (BEA) also negatively affect oocyte development [2,6]. Deoxynivalenol is a *Fusarium* mycotoxin able to bind to ribosomes, inducing ribotoxic stress responses [9], which inhibit translation and activate mitogen-activated protein (MAP) kinases [10], resulting in cell apoptosis, increased release of reactive oxygen species (ROS), and the production of superoxide by mitochondria [11]. Beauvericin is another *Fusarium* mycotoxin commonly present in grains contaminated with DON. BEA can exhibit antibacterial, antiviral, antifungal, antiparasitic, insecticidal, and anticarcinogenic activities [12,13]. These effects may be related to the ability of BEA to induce oxidative stress [14].

Healthy cells are constantly maintaining a balance between ROS production and antioxidant activity to guarantee the redox balance. The same mechanism is observed in gametes. Reactive oxygen species serve as signal molecules playing a regulatory role in oocyte maturation, but excess of ROS needs to be neutralized by antioxidants to protect the oocyte and the subsequently produced embryo [15]. Antioxidants produced by cells (enzymatic) or acquired via the diet (non-enzymatic) will scavenge ROS by their conversion into water and alcohol molecules. The responsible enzymatic antioxidants are superoxide dismutase (SOD), catalase (CAT), glutathione peroxidase (GPx), and glutathione reductase (GSR), whereas non-enzymatic antioxidants include vitamins and glutathione (GSH), among others [16]. In addition, when cells are challenged with oxidative stress, various chaperones can be activated, like the heat shock protein 70 (HSP70) to assure protein folding and normal cell function [17], whereas an overproduction of ROS will increase the expression of the hypoxia inducible factor (HIF1A) [18]. The relationship of these two markers with mycotoxin exposure was previously demonstrated [19,20].

In vitro maturation is less efficient with oocytes from gilts compared with oocytes from sows [21]. The reduced capacity of gilt oocytes to properly mature in vitro is related to their limited exposure to hormones [22,23], but also because they are more susceptible to oxidative stress [24]. In pre-pubertal gilts, the endocrine signals to initiate puberty allow the growth of follicles up to ~6 mm, with a subsequent high atresia rate, and the first estrus of gilts is classified as a heat-no-serve, not being considered for insemination [25]. Yuan et al. [24] demonstrated that prepubertal oocytes cannot cope with the redox balance as efficiently as oocytes from adult sows, particularly because of decreased expression of glutaredoxin 2 (GLRX2), protein disulfide isomerase 4 (PDIA4), and thioredoxin reductase (TXNRD1), and increased glutathione (GSH) content in gilt oocytes. However, the surrounding cumulus cells and follicular fluid (FF) are also important. The FF is initially produced by granulosa cells surrounding the immature oocyte, and this fluid will fill the antral cavity and contains hormones, growth factors, nutrients, reactive oxygen species (ROS), and antioxidants [26]. In antral follicles, the communication between oocyte and cumulus cells in the cumulus-oocyte complexes (COCs) allows the cytoplasmic and nuclear maturation and fertilization of the oocyte [27]. Both of these compartments count on a redox system to maintain oocyte quality, via enzymatic and non-enzymatic antioxidant defense [28].

The metabolism and transcription in oocytes from prepubertal gilts are not similar to those in cycling sows, affecting the development potential [29], and this might also be related to oxidative stress. Based on this, we hypothesize that oocytes from gilts and sows will respond differently to DON and BEA exposure during in vitro maturation, and that the follicular redox system plays an important role in these differences. In the present study, we evaluated the effect of BEA, DON, and the ROS hydrogen peroxide (H_2_O_2_) at different concentrations on in vitro maturation of oocytes from gilts and sows. Furthermore, antioxidant levels in the FF, oocytes, and cumulus cells were established, as well as the relative mRNA expression of redox markers.

## 2. Results

### 2.1. Oocytes from Gilts Are More Sensitive to H_2_O_2_, BEA, and DON during Maturation Than Those from Sows

Oocytes from gilts and sows were exposed to a reactive oxygen species (H_2_O_2_) and two *Fusarium* mycotoxins (BEA and DON) during in vitro maturation. A significant decrease in the rate of nuclear maturation was observed when oocytes from gilts were in vitro matured in the presence of 50 or 100 μmol/L H_2_O_2_, whereas oocytes from sows were unaffected. Moreover, the degeneration rate of gilt oocytes significantly increased after maturation in the presence of 50 or 100 μmol/L H_2_O_2_, and of sow oocytes in the presence of 100 μmol/L H_2_O_2_. For both maturation and degeneration rates, there was a significant linear effect with the increase of the H_2_O_2_ concentration in the maturation medium (Table 1).

A significant decrease in the rate of nuclear maturation was observed with gilt oocytes already in the presence of 0.5 μmol/L BEA, and this negative impact was even more prominent at concentrations of 2.5 or 5.0 μmol/L. Differently, BEA did not affect the maturation of sow oocytes at the tested concentrations when compared to control. Likewise, the degeneration rate of gilt oocytes was significantly increased in the presence of BEA regardless of the tested concentration, while BEA did not significantly alter the degeneration of sow oocytes. For both maturation and degeneration rates, there was a significant linear effect with an increase of the BEA concentration in the maturation medium (Table 2).

Regardless of the age (gilts or sows), a significant decrease in the rate of nuclear maturation was observed when oocytes were in vitro matured in the presence of 0.02 or 0.2 μmol/L DON, and this negative impact was even more prominent at a concentration of 2.0 μmol/L. Also, gilt oocytes were more sensitive to DON exposure than sow oocytes. Regarding the degeneration rate, no effect of DON exposure was observed. A significant linear effect with an increase of the DON concentration in the maturation medium was observed only when evaluating maturation rate (Table 3).

### 2.2. Oxidative Stress in Oocytes and Cumulus Cells Depends on the Maturation Stage and Donor Age, i.e., Gilts or Sows

To understand the higher sensitivity to stressors of oocytes from gilts than from sows, markers of oxidative stress were evaluated before and after in vitro maturation.

**Figure 1:**
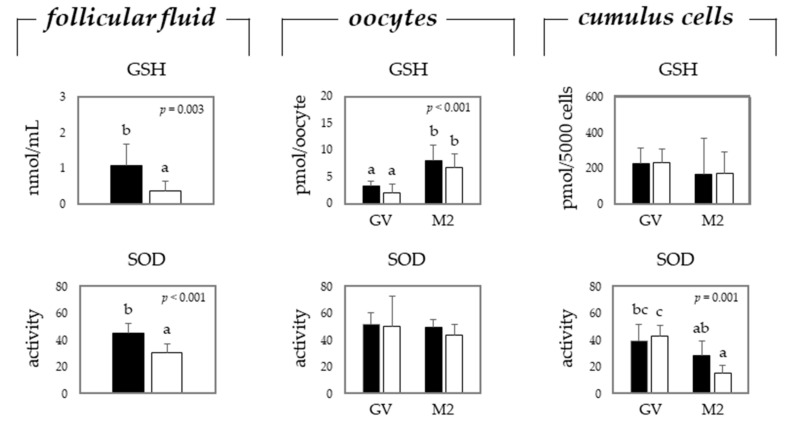
Mean (±SEM) levels of glutathione (GSH) and superoxide dismutase (SOD) in the follicular fluid, oocytes, and cumulus cells from gilts (black bars) and sows (white bars). Oocytes and cumulus cells were analyzed before (germinal vesicle (GV) stage) and after (metaphase 2 (M2) stage) in vitro maturation. Each mean represents values from 5 replicates per gilt or sow (pooled follicular fluid from antral follicles measuring 2–8 mm, and 50 COCs/replicate/gilt or sow), using the animal as the experimental unit. a–c: different letters indicate significant difference among treatments, i.e., gilts vs. sows for follicular fluid, and gilts vs. sows and GV vs. M2 for oocytes and cumulus cells (*p* ≤ 0.05).

The total equivalent antioxidant capacity (TEAC) in the follicular fluid was determined, but no differences were observed (data not shown). Glutathione (GSH) and superoxide dismutase (SOD) activity were significantly higher in the FF from gilts than from sows. When evaluating oocytes before (GV stage) or after maturation (M2 stage), no differences were observed when comparing gilts with sows. It was remarkable that GSH levels significantly increased in mature oocytes, while the SOD activity had significantly decreased in their cumulus cells. Furthermore, the SOD activity had significantly decreased in the cumulus cells from mature sow oocytes, whereas this was not observed in mature oocytes from gilts (Figure 1).

**Figure 2:**
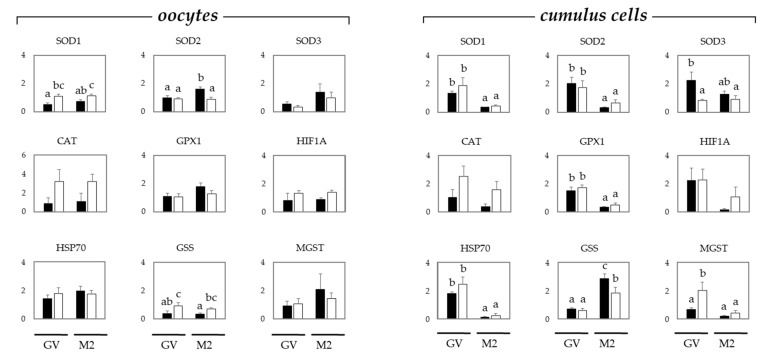
Mean (±SEM) mRNA relative expression of markers for oxidative stress in oocytes, and cumulus cells from gilts (black bars) and sows (white bars). Gene abbreviations are above each graph, full names of the different genes are presented in the main text. Oocytes and cumulus cells were analyzed before (GV stage) and after (M2 stage) in vitro maturation. Each mean represents values from 5 replicates per gilt or sow (50 COCs/replicate/gilt or sow), using the animal as the experimental unit. a–c: different letters indicate significant difference among treatments, gilts vs. sows and GV vs. M2 (*p* ≤ 0.05).

To study the oxidative stress in oocytes and cumulus cells, gene expression levels were examined in COCs by quantitative reverse transcription-polymerase chain reaction (qRT-PCR) (Figure 2).

In oocytes, the relative expression of *SOD1* was not affected by the maturation stage, but it was significantly higher in sows than in gilts. The relative expression of *SOD2* was significantly increased only in mature oocytes from gilts, whereas no changes were observed in the expression of *SOD3*. The relative expression of glutathione synthetase (*GSS*) was significantly higher in the oocytes from sows than in those from gilts, regardless of the maturation stage. The relative expression of catalase (*CAT*), glutathione peroxidase 1 (*GPX1*), hypoxia-inducible factor 1 alpha (*HIF1A*), heat shock protein 70 (*HSP70*), and microsomal glutathione S-transferase (*MGST*) was similar in the oocytes from gilts and sows, regardless of the maturation stage (GV or M2).

The expression of *SOD1*, *SOD2*, *GPX1*, and *HSP70* in cumulus cells was significantly decreased after maturation regardless of the donor age, i.e., gilts or sows. The relative expression of *SOD3* and *MGST* was significantly the highest in the cumulus cells from immature COCs from gilts and sows, respectively. The relative expression of GSS was significantly higher in the cumulus cells from mature oocytes from gilts than in those from sows. The relative expression of *CAT* and *HIF1A* was similar in the cumulus cells from gilts and sows, regardless of the maturation stage (GV or M2).

## 3. Discussion

In the present study, we demonstrated that oocytes from gilts are more sensitive than those from sows when exposed to DON or BEA during in vitro maturation, and this effect is influenced by the redox balance in these gametes. Previous studies showed that the maturation of oocytes from sows is impaired when performed in the presence of 0.2 μmol/L DON [4].

Herein, a decrease in the maturation rate was already observed after exposure of oocytes to 0.02 μmol/L DON, and this decrease was more evident in the oocytes from gilts. Han et al. [3] also demonstrated the negative impact of DON on the maturation of gilt oocytes at relatively high concentrations of this mycotoxin: the maturation rate decreased to 55% after exposure to 2.0 μmol/L DON and to less than 10% when exposed to 3.0 μmol/L DON. Disruption of oocyte maturation was most likely caused by an increased rate of apoptosis and epigenetic modification after exposure to 3.0 μmol/L DON [3]. In another study, malformation of meiotic spindles were observed after exposure to 0.2 and 2.0 μmol/L DON, with a decrease in the maturation rate of maturation rate to 25% when the oocytes were exposed to 2.0 μmol/L DON [4]. The sensitivity of oocytes to oxidative stress during maturation was also demonstrated after exposure to H_2_O_2_ when oocytes from gilts were the most affected.

DON does not affect the glutathione levels in oocytes [4], most likely since the cumulus cells protect oocytes by synthesizing and transporting GSH to oocytes [30,31]. In the present study, GSH levels in the oocytes and cumulus cells were not affected by the donor age (gilts or sows), but by the maturation stage in oocytes. Likewise, Pawlak et al. [32] observed an increase in the GSH content in matured porcine oocytes, regardless of the donor (prepubertal or cycling gilt). In the present study, the GSH content was significantly the highest in gilt FF. FF is a plasma-like fluid containing metabolites, proteins, ROS, and antioxidants from COCs, but also plasma products including antioxidants [33], where the GSH and SOD levels in FF are usually similar to those in the serum [34]. The GSH content in FF is not related to follicular size, but its presence will protect the oocytes against oxidation during growth and maturation [35], although there is no correlation between GSH and steroids in FF [36]. A decrease in the SOD activity in FF has been correlated with follicular growth in pigs [37] and high fertility rates in human [38]. It seems, however, that GSH and SOD levels in the FF do not predict resistance to toxins. Furthermore, the antioxidant capacity of granulosa cells exposed to AOH was increased when these cells were cultured in the presence of FF from sows when compared with FF from gilts [39]. Also, regardless of the FF source, no protection was observed when granulosa cells were exposed to BEA, showing that the FF alone cannot cope with the stress caused by this mycotoxin [39]. The ability of SOD to inhibit ROS activity in the cumulus cells was significantly decreased after maturation of oocytes from sows, but this effect was not observed in the cumulus cells from gilts. Increased SOD activity can be an indicator of oxidative stress, which could explain why gilt oocytes were more sensitive to the stress conditions during in vitro maturation. Such effects were even more robust when gilt oocytes were matured in the presence of BEA, and an interaction with dose and animal age was observed. There was no effect of BEA on the nuclear maturation when oocytes were cultured in the presence of BEA up to 5.0 μmol/L. However, a ten-fold lower concentration was sufficient to impair the maturation of gilt oocytes, and all oocytes degenerated after exposure to 5.0 μmol/L BEA. In a previous study, oocytes from gilts presented a significant decrease in maturation only when exposed to 2.5 μmol/L BEA [5]. However, the oocytes were cultured in a medium containing sow FF and not gilt FF as in the present study.

Interestingly, exposure to ROS (H_2_O_2_) also resulted in an impaired maturation rate in gilt oocytes, indicating that the intrinsic redox balance from gilt oocytes is different from that of sow oocytes. To understand the differences between gametes from gilts and sows, the mRNA expression of markers for oxidative stress was evaluated in oocytes and cumulus cells before and after in vitro maturation.

The SOD enzymes metabolize the ROS O_2_^−^ into H_2_O_2_ and O_2_, and different enzymes can be categorized: SOD1, Cu/ZnSOD is cytoplsmaic, SOD2, MnSOD is mitochondrial, and SOD3 with a structure homologous to SOD1 [40] is secreted by cells. The expression of *SOD1* was significantly higher in sows’ oocytes regardless of the maturation stage. The gene expression increase of this cytosolic SOD indicates that oocytes from sows are more active in neutralizing superoxide anions in the cytoplasm, whereas the increase of *SOD2* in mature gilts’ oocytes is expected because oocytes derived from small follicles of slaughterhouse ovaries also express high levels of SOD2 [41]. However, this may also reflect an incorrect degradation of maternal mRNA prior to the activation of the embryonic genome [42]. The *SOD3* expression in oocytes and cumulus cells from gilts and sows was similar within each maturation stage, except for the cumulus cells from immature oocytes that presented a *SOD3* upregulation. This SOD is responsible for scavenging O_2_^−^ in extracellular spaces and may explain why the FF from gilts had a significantly higher activity of this antioxidant. A proper functioning of the antioxidant enzymes SOD and CAT is crucial when cells are exposed to BEA [43]. Deoxynivalenol disrupts the normal function of mitochondria, affecting the normal function of SOD2 and increasing the accumulation of ROS in the cells [44]. In the present study, CAT relative expression was not affected by the COCs’ donor age and the imbalance in SOD expression probably explains the decreased maturation rate of gilt oocytes exposed to BEA and DON.

The relative expression of *GPX1* and *HSP70* was related to oocyte maturation and observed only in cumulus cells. Microsomal glutathione S-transferase was upregulated in immature oocytes from sows. The expression of *MGST* is increased in steroidogenically active cells [45], explaining the present results. Oocytes from sows had a higher expression of *GSS* than gilts, regardless of the maturation stage, whereas this effect was not observed in cumulus cells, which had a high expression of GSS when harvested from in vitro matured gilt oocytes. It has been demonstrated that GSS expression is higher in oocytes from sheep better able to develop to blastocysts [46]. Also, oocytes from prepubertal mice have a decreased capacity to synthesize GSH, increasing the chances of oocyte damage via oxidative stress [47]. Although oocytes and cumulus cells are connected, a completely different picture was observed in cumulus cells, where maturation was characterized by an upregulation of GSS, especially in those from matured gilt oocytes that present a lower glucose metabolism than sows, resulting in a decrease in GSH-S-transferase [48]. Cumulus cells synthesize GSH during oocyte maturation to guarantee fertilization and embryo development [49]. Possibly, in gilts, this was a compensatory mechanism due to the low GSS expression in mature oocytes. Both DON [50] and BEA [51] induce oxidative stress and also decrease the capacity to synthesize GSH, partly explaining the high susceptibility of gilt COCs to these mycotoxins during maturation.

When studying the potential effects of mycotoxins on female reproduction, it is crucial to consider that exposure via dietary contamination will give a more accurate model to predict risks [52]. Herewith, we show that the age of the donors will also affect the toxicity results, indicating that prepubertal females are more sensitive to mycotoxin exposure. Pigs are appropriate models to determine the effects of toxic compounds in humans due to the physiological similarities [53]. Therefore, chronic dietary exposure of children and prepubertal girls to DON and BEA may affect their fertility in adult life, and these mycotoxins are commonly found in infant food [54]. The tested exposure levels of DON in the present studies are similar to those detected in porcine and human plasma. The plasma level in pigs chronically exposed to DON reached 0.09 μmol/L [55], whereas DON was detected in the urine from healthy consumers at levels up to 1.25 μmol/L [56]. The detection of BEA in biological samples is a complex process because this mycotoxin is present in the diet at far lower concentrations than DON and is rapidly metabolized to a range of yet uncharacterized derivatives [57].

## 4. Conclusions

In conclusion, oocytes from gilts are more susceptible to the toxic effects of DON and BEA when compared with oocytes from sows. This effect is observed when immature oocytes start their maturation and the redox balance in sows is better controlled that in gilt oocytes and are mostly related to the imbalance of SOD and GSH in oocytes. Both DON and BEA do not affect cell viability solely via the stimulation of oxidative stress, but their negative impact will be potentiated in cells that cannot properly scavenge metabolic products like ROS.

## 5. Materials and Methods

### 5.1. Chemicals

Chemicals were purchased from Sigma (St. Louis, MO, USA) unless stated otherwise. BEA (purity > 97%) was dissolved in dimethyl sulfoxide (DMSO) to obtain a stock solution of 20 mM. Aliquots of these stock solutions were stored at −20 °C. On the day of experiment, the stock solution was diluted in culture medium and sonicated for 15 min to reach the concentration of 50 μM. DON (purity > 97%) was dissolved in DMSO to obtain a stock solution of 20 mM. Aliquots of these stock solutions were stored at −20 °C. On the day of experiment, the DON stock solution was diluted in culture medium and sonicated for 15 min to reach the concentration of 20 μM. The final concentration of DMSO in the treatment medium was 0.02% *v/v*. Therefore, medium used as control also had 0.02% DMSO. Culture media were equilibrated at 5% CO_2_ and 38.5 °C for at least two hours before use.

### 5.2. Selection and Culture of Cumulus Oocyte Complexes

Collection and selection of porcine cumulus oocyte complexes (COCs) were performed as described [58]. Briefly, ovaries from prepubertal gilts and sows collected from a local slaughterhouse were transported to the laboratory and maintained at 30 °C. Antral follicles (2–6 mm in diameter) were aspirated and COCs with a compact cumulus mass were selected, transferred to 4-(2-hydroxyethyl)-1-piperazineethanesulfonic acid (HEPES)-buffered M199 (Gibco BRL, Paisley, UK) supplemented with penicillin and streptomycin, and washed 3 times in oocyte maturation medium (OMM) consisting of M199 supplemented with 2.2 mg/mL NaHCO_3_, 200 μM cysteamine, with or without 10% (*v/v*) sow follicular fluid. Follicular fluid was collected from sow ovarian follicles measuring 5–8 mm in diameter. COCs were cultured for 20–22 h at 38.5 °C in a humidified atmosphere of 5% CO_2_ in air in groups of 35–50 in 500 μL of OMM with 0.05 international units (IU)/mL recombinant human follicle stimulating hormone (FSH) (Organon, Oss, The Netherlands), followed by a 20–22 h culture period in OMM without FSH.

### 5.3. Assessment of Cumulus Oocyte Complex Viability and Oocyte Nuclear Maturation

At 44 h of culture, COCs were incubated with 2 μM Ethidium Homodimer-1 (EtHD-1; Invitrogen-Molecular Probes, Eugene, OR, USA) in OMM and cultured for another 4 h. COCs were washed 3 times in phosphate buffered saline (PBS) and fixed in 2% (*w/v*) formaldehyde. Subsequently, COCs were incubated for 5 min in 0.1 μg/mL 4,6-diamino-2-phenyl-indole (DAPI; Invitrogen-Molecular Probes, Landsmeer, the Netherlands), mounted under a coverslip with anti-fade mounting medium (Vectashield; Vectorlab, Burlingame, CA, USA), and imaged by confocal laser scanning microscopy (CLSM; Leica SPE-II, Heidelberg, Germany). Imaging was performed using a 368 and 543 nm laser to excite DAPI and EthD-1, respectively. Nuclei of cumulus cells were considered degenerated when stained for both DAPI and EthD-1, or when nuclei had a condensed or fragmented appearance. Viable oocytes containing a metaphase spindle together with a polar body were considered at the Metaphase II (M2) stage.

### 5.4. Markers of Antioxidant Capacity and Oxidative Stress

Total equivalent antioxidant capacity (TEAC) in the FF (mM) was measured by using an assay kit from Sigma (Saint Louis, MO, USA; no. CS0790). Glutathione (GSH) levels in the FF (nmol/mL) and oocytes (relative fluorescence units (RFU)/min) was measured by using a kit from BioVision (Milpitas, CA, USA; no. K740-100). Hydrogen peroxide (H_2_O_2_) in FF (nmol/mL) and oocytes (pmol/oocyte) was measured by using a kit from BioVision (no. K265-200). Superoxide dismutase (SOD) activity (% of inhibition) in the FF, oocytes, and cumulus cells was measured by using an assay kit from BioVision (no. K335-100).

### 5.5. Quantitative Reverse Transcription-PCR

Total RNA was extracted with an RNAeasy Mini Kit (Qiagen, Valencia CA, USA) as per manufacturer’s instructions. Total RNA (10 μL) was kept at 70 °C for 5 min and then chilled on ice. Ten μL of a mastermix containing 4 μL 5 × 1st strand buffer, 0.4 μL random primers (0.09 IU/mL), 0.2 μL RNAsin (40 IU/mL), 0.75 μL Superscript^®^III (200 IU/mL) (Invitrogen, Groningen, The Netherlands), 2 μL dithiothreitol (0.1 M), 1 μL deoxyribose nucleoside triphosphate (dNTP) mix (10 mM), and 1.65 μL H_2_O were added and the mixture was incubated at 50 °C for 1 h. As a negative control, reverse transcriptase was replaced by H_2_O (minus reverse transcriptase (-RT) blanks). Samples were subsequently kept at 70 °C for 15 min and stored at −20 °C. The PCR mixture contained 0.1 μL cDNA, 0.1 μL forward and reverse primers (100 μM each) (Isogen, Maarssen, The Netherlands), 10 μL iQ SYBR Green supermix (Bio-Rad Laboratories, Hercules, CA, USA), and 9.8 μL H_2_O. The oligonucleotide primers were designed using Primer Select software (DNAstar, Madison WI, USA), and amplification profiles are listed in Table 4. After an initial denaturation step at 95 °C for 3 min, 40 cycles were carried out consisting of 95 °C for 5 s, the primer-specific annealing temperature (Table 4) for 5 s, and 72 °C for 20 s. Quantitative RT-PCR was performed in duplicate on two replicates of cDNA and singular on the -RT blanks. For each target gene, all the samples were quantified simultaneously in one run in a 96-well plate using a real-time PCR detection system (MyiQ Single-color, Real-Time Detection System; Bio-Rad). Melting curves were plotted to verify single product amplification. Standard curves made on cDNA dilutions were used to calculate the relative starting quantities. RNA expression was calculated as the ratio of RNA starting quantity of the target gene/geometric mean of two reference genes (*BACT* and *B2M*, cumulus; *GAPDH* and *PGK1*, oocytes).

### 5.6. Statistical Analysis

The COCs from gilts or sows were randomly assigned to treatment conditions prior to each experiment. Statistical analysis was conducted with the GenStat statistical software (GenStat for Windows 20th Edition, VSN International, Hemel, Hempstead, UK; https://www.vsni.co.uk/downloads/genstat/, accessed on 5 April 2021). Data were compared with analysis of variance (ANOVA). The null hypothesis was that there was no treatment effect on the response parameter. Treatment means were compared with Tukey’s post-hoc test, where the sow or gilt was the experimental unit. Linear and quadratic regression were applied to assess the effects of H_2_O_2_, BEA, and DON concentrations on oocyte maturation and degenerations. Data are presented as mean ± standard error of the mean (SEM). The *p*-value of the statistical model is given per response parameter. Significance was declared at *p* ≤ 0.05.

## Figures and Tables

**Table 1:** Mean (±standard error of the mean (SEM)) rates of nuclear maturation and degeneration of porcine in vitro matured in the absence (control) or in the presence of H_2_O_2_ at different concentrations.

Treatment	Age	Maturation Rate		Degeneration Rate	
Control	Gilt	82.9	c	4.8	a
Control	Sow	86.9	c	2.4	a
10 μmol/L	Gilt	65.7	c	9.1	a
10 μmol/L	Sow	86.8	c	2.5	a
50 μmol/L	Gilt	35.7	b	26.8	b
50 μmol/L	Sow	71.9	c	9.7	a
100 μmol/L	Gilt	7.7	a	88.1	c
100 μmol/L	Sow	65.5	c	30.4	b
Control		84.9		3.6	
10 μmol/L		76.2		5.8	
50 μmol/L		53.8		18.2	
100 μmol/L		36.6		59.2	
	Gilt	48.0		32.2	
	Sow	77.8		11.2	
Effect of:		*p*-value	SEM	*p*-value	SEM
Treatment × Age		0.017	7.60	<0.001	3.34
Age		<0.001	3.80	<0.001	1.67
Treat		<0.001	5.38	<0.001	2.36
Treatment Linear		0.006		<0.001	
Treatment Quadratic		0.494		0.072	

Each mean represents values from 5 replicates per gilt or sow (50 cumulus oocyte complexes (COCs)/replicate/gilt or sow), using the animal as the experimental unit. a–c: different letters indicate significant difference among treatments (*p* ≤ 0.05).

**Table 2:** Mean (± SEM) rates of nuclear maturation and degeneration of porcine in vitro matured in the absence (control) or in the presence of BEA at different concentrations (0.5, 2.5, or 5.0 μmol/L).

Treatment	Age	Maturation Rate		Degeneration Rate	
Control	Gilt	74.0	cd	11.7	a
Control	Sow	77.4	cd	1.5	a
0.5 μmol/L	Gilt	46.8	b	28.1	b
0.5 μmol/L	Sow	82.0	d	2.7	a
2.5 μmol/L	Gilt	4.8	a	91.6	c
2.5 μmol/L	Sow	72.3	cd	9.7	a
5.0 μmol/L	Gilt	0.0	a	100.0	c
5.0 μmol/L	Sow	64.8	c	11.1	a
Control		75.7		6.6	
0.5 μmol/L		64.4		15.5	
2.5 μmol/L		38.6		50.7	
5.0 μmol/L		32.4		55.5	
	Gilt	31.4		57.9	
	Sow	74.1		6.2	
Effect of:		*p*-value	SEM	*p*-value	SEM
Treatment × Age		<0.001	5.08	<0.001	3.59
Age		<0.001	2.54	<0.001	1.80
Treat		<0.001	3.59	<0.001	2.54
Treatment Linear		0.016		0.010	
Treatment Quadratic		0.857		0.319	

Each mean represents values from 5 replicates per gilt or sow (50 COCs/replicate/gilt or sow), using the animal as the experimental unit. a–d: different letters indicate significant difference among treatments (*p* ≤ 0.05).

**Table 3:** Mean (± SEM) rates of nuclear maturation and degeneration of porcine in vitro matured in the absence (control) or in the presence of DON at different concentrations (0.02, 0.2, or 2.0 μmol/L).

Treatment	Age	Maturation Rate		Degeneration Rate	
Control	Gilt	67.5		13.3	
Control	Sow	73.2		9.1	
0.02 μmol/L	Gilt	37.1		12.7	
0.02 μmol/L	Sow	63.5		7.5	
0.2 μmol/L	Gilt	22.9		29.7	
0.2 μmol/L	Sow	59.7		8.8	
2.0 μmol/L	Gilt	8.7		35.4	
2.0 μmol/L	Sow	42.5		7.5	
Control		70.4	c	11.2	
0.02 μmol/L		50.3	b	10.1	
0.2 μmol/L		41.3	b	19.2	
2.0 μmol/L		25.6	a	21.5	
	Gilt	34.0	a	22.8	b
	Sow	59.7	b	8.2	a
Effect of:		*p*-value	SEM	*p*-value	SEM
Treatment × Age		0.178	7.17	0.089	5.04
Age		<0.001	3.58	0.001	2.52
Treat		<0.001	5.07	0.101	3.57
Treatment Linear		0.026		0.798	
Treatment Quadratic		0.863		0.885	

Each mean represents values from 5 replicates per gilt or sow (50 COCs/replicate/gilt or sow), using the animal as the experimental unit. a–c: different letters indicate significant difference among treatments (*p* ≤ 0.05).

**Table 4:** Primer pairs sequence used in polymerase chain reaction (PCR) to detect the primary partial cDNA products in porcine oocytes and cumulus cells.

Gene	Protein	Sequence	Product Size (bp)	Annealing Temperature (°C)	Gene Bank Accession Number
**Reference**					
*B2M*	β-2-microglobulin	F: 5′-TTCACACCGCTCCAGTAG-3′ R: 5′-CCAGATACATAGCAGTTCAGG-3′	166	60.0	NM_213978
*BACT*	β-Actin	F: 5′-CATCACCATCGGCAACGAGC-3′ R: 5′-TAGAGGTCCTTGCGGATGTC-3′	141	56.0	AY550069
*PGK1*	Phosphoglycerate kinase 1	F: 5′-AGATAACGAACAACCAGAGG-3′ R: 5′-TGTCAGGCATAGGGATACC-3′	126	56.0	AY677198
*GAPDH*	Glyceraldehyde 3-phosphate dehydrogenase	F: 5′ -TCGGAGTGAACGGATTTG-3′ R: 5′-CCTGGAAGATGGTGATGG-3′	219	61.0	AF017079
***Target***					
*CAT* [59]	Catalase	F: 5′-ATGTGCAGGCTGGATCTCAC-3′ R: 5′-GCACAGGAGAATCTTGCATCC-3′	155	55.0	XM_021081498.1
*GPX1* [60]	Glutathione peroxidase 1	F: 5′-CAAGAATGGGGAGATCCTGA-3′ R: 5′-GTCA TTGCGACACACTGGAG-3′	217	64.5	NM_2142201.1
*GSS*	Glutathione synthetase	F: 5′-TGGTTTACTTCCGGGATGGC-3′ R: 5′-CGCCTACTCTGCTTAGCTCC-3′	159	64.5	NM_001244625.1
*HIF1A* [61]	Hypoxia inducible factor 1 alpha	F: 5′-TCAGCTATTTGCGTGTGAGG-3′ R: 5′-TTCACAAATCAGACCAAGC-3′	479	61.0	NM_001123124.1
*HSP70* [60]	Heat shock protein 70	F: 5′-ATGTCCGCTGCAAGAGAAGT-3′ R: 5′-GGCGTCAAACACGGTATTCT-3′	216	64.5	NM_001123127.1
*MGST*	Microsomal glutathione S-transferase 1	F: 5′-CGAGGAATTAAGATAGAGAAAGCCT-3′ R: 5′-TGGGCCCATTTGGAAAATACTGA-3′	247	64.5	NM_214300.2
*SOD1* [59]	Superoxide dismutase 1	F: 5′-CGAGCTGAAGGGAGAGAAGA-3′ R: 5′-ACATTGCCCAGGTCTCCAAC-3′	199	64.5	NM_001190422.1
*SOD2* [61]	Superoxide dismutase 2	F: 5′-CCCTGGAGCCGCACATC-3′ R: 5′-TTTTTCAGCGCCTCCTG-3′	115	64.5	NM_214127.2
*SOD3* [62]	Superoxide dismutase 3	R: 5′-ACTCCTGCCATGCTGACG-3′ F: 5′-TGCCAGATCTCCGTCACTTT-3′	140	64.5	DQ915492.1

## Data Availability

Data sharing not applicable.

## Abbreviations

ANOVA	Analysis of variance
AOH	Alternariol
B2M	β-2-microglobulin
BACT	β-Actin
BEA	Beauvericin
CAT	Catalase
cDNA	Complement deoxyribonucleic acid
COCs	Cumulus oocyte complexes
Cu/ZnSOD	Copper/zink superoxide dismutase
DAPI	4′,6-diamidino-2-phenylindole
DMSO	Dimethyl sulfoxide
dNTP	Deoxyribonucleotide triphosphate
DON	Deoxynivalenol
EthD-1	Ethidium homodimer-1
FF	Follicular fluid
FSH	Follicle-stimulating hormone
GAPDH	Glyceraldehyde 3-phosphate dehydrogenase
GLRX2	Glutaredoxin 2
GPx	Glutathione peroxidase
GPX1	Glutathione peroxidase 1
GSH	Glutathione
GSR	Glutathione reductase
GSS	Glutathione synthetase
GV	Germinal vesicle
HEPES	4-(2-hydroxyethyl)-1-piperazineethanesulfonic acid
HIF1A	Hypoxia-inducible factor 1
HSP70	Heat shock protein 70
IU	International units
M2	Metaphase II
MGST	Microsomal glutathione S-transferase
MnSOD	Manganese superoxide dismutase
OMM	Oocyte maturation medium
PBS	Phosphate buffer saline
PCR	Polymerase chain reaction
PDIA4	Protein disulfide isomerase 4
PGK1	Phosphoglycerate kinase 1
qRT-PCR	quantitative reverse transcription-polymerase chain reaction
RFU	Relative fluorescence units
RNA	Ribonucleic acid
ROS	Reactive oxygen species
-RT	Minus reverse transcriptase
SEM	Standard error of the mean
SOD	Superoxide dismutase
SOD1	Superoxide dismutase 1
SOD2	Superoxide dismutase 2
SOD3	Superoxide dismutase 3
TEAC	Total equivalent antioxidant activity
TXNRD1	Thioredoxin reductase
ZEN	Zearalenone
